# Aneuploidy in pluripotent stem cells and implications for cancerous transformation

**DOI:** 10.1007/s13238-014-0073-9

**Published:** 2014-06-05

**Authors:** Jie Na, Duncan Baker, Jing Zhang, Peter W. Andrews, Ivana Barbaric

**Affiliations:** 1 School of Medicine, Tsinghua University, Beijing, 100084 China; 2 Sheffield Diagnostic Genetic Services, Sheffield Children’s Hospital, Sheffield, S10 2TH UK; 3 Centre for Stem Cell Biology, Department of Biomedical Science, The University of Sheffield, Sheffield, S10 2TN UK

**Keywords:** human pluripotent stem cells (hPSCs), culture adaptation, aneuploidy, cancer, genetic changes

## Abstract

Owing to a unique set of attributes, human pluripotent stem cells (hPSCs) have emerged as a promising cell source for regenerative medicine, disease modeling and drug discovery. Assurance of genetic stability over long term maintenance of hPSCs is pivotal in this endeavor, but hPSCs can adapt to life in culture by acquiring non-random genetic changes that render them more robust and easier to grow. In separate studies between 12.5% and 34% of hPSC lines were found to acquire chromosome abnormalities over time, with the incidence increasing with passage number. The predominant genetic changes found in hPSC lines involve changes in chromosome number and structure (particularly of chromosomes 1, 12, 17 and 20), reminiscent of the changes observed in cancer cells. In this review, we summarize current knowledge on the causes and consequences of aneuploidy in hPSCs and highlight the potential links with genetic changes observed in human cancers and early embryos. We point to the need for comprehensive characterization of mechanisms underpinning both the acquisition of chromosomal abnormalities and selection pressures, which allow mutations to persist in hPSC cultures. Elucidation of these mechanisms will help to design culture conditions that minimize the appearance of aneuploid hPSCs. Moreover, aneuploidy in hPSCs may provide a unique platform to analyse the driving forces behind the genome evolution that may eventually lead to cancerous transformation.

## INTRODUCTION

The goal of regenerative medicine is to enhance the healing potential of the body or replace damaged tissues and organs. This presents an immense challenge, but the basic concepts and the technology appear poised to deliver this aim. Central to these efforts are human pluripotent stem cells (hPSCs), including embryonic stem cells (hESCs) derived from human blastocysts (Thomson et al., 1998) and induced pluripotent stem cells (hiPSCs) generated through reprogramming of differentiated cells (Takahashi et al., 2007), as they can give rise to any cell type of the body. However, the barriers along the translational pipeline are still numerous. Firstly, an essential prerequisite for using hPSCs in clinical applications is the maintenance of large numbers of homogeneous, undifferentiated stem cells in culture. Yet, spontaneous appearance of genetic and epigenetic variants contributes to the phenotypic diversity of the individual cultures. Furthermore, the existence of variant cells creates an essential substrate for *in vitro* selection whereby mutations that endow cells with improved growth outcompete their normal counterparts and overtake the culture—a phenomenon termed *culture adaptation* (Baker et al., 2007; Enver et al., 2005). The mutational diversification and clonal selection of hPSCs in culture is an inevitability of basic evolutionary principles. However, the presence of genetic changes in hPSCs coupled with their increased growth rates is reminiscent of the defining features of cancer cells (Baker et al., 2007). Viewed in this light, genetic changes are tempering hope for the safe use of hPSCs in medicine.

The occurrence of non-random genetic changes in hPSC cultures is now well established, but the genetic roadmap that leads to the complex mutations remains obscure. With hPSCs entering clinical trials (Schwartz et al., 2012), the need to identify driver mutations underpinning the culture adaptation is particularly pressing. In addition to the clinical relevance, hidden within the complex mutational profiles are clues to the basic mechanisms governing stem cell fates. Here, we provide an overview of the types of genetic changes commonly observed in hPSC cultures and their functional consequences for hPSC phenotype and behavior. Furthermore, we discuss the putative cellular mechanisms underpinning the generation of the observed mutations. Finally, we draw parallels between the genetic changes observed in hPSCs with the ones commonly detected in human cancers and early development, as integration of this information will facilitate efforts to pinpoint the candidate genes, molecular mechanisms and environmental factors driving the culture adaptation.

## GENETIC CHANGES IN hPSCs DURING *IN VITRO* CULTURE

HESCs originate from the inner cell mass of early human blastocysts where they exist only during a short window of embryo development, prior to differentiating into the cells of all three embryonic germ layers (Murry and Keller, 2008). Placing the inner cell mass *in vitro* under the finely tuned culture conditions prevents their imminent differentiation and allows cells to self-renew seemingly indefinitely whilst retaining their differentiation potential (Thomson et al., 1998). The shift from the *in vivo* niche to the life in an *in vitro* environment is accompanied by marked transcriptional changes (Yan et al., 2013) and is undoubtedly a stressful event for cells. Conceivably, this may act as a trigger for genome changes akin to (epi)genetic alterations associated with the tissue culture in plants (McClintock, 1984). Nonetheless, at least at the gross karyotype level, the majority of hESC lines are normal upon derivation (Amps et al., 2011; Thomson et al., 1998).

The production of hiPSCs by reprogramming of somatic cells is a sequential process that starts by obtaining somatic cells and placing them *in vitro*, followed by genetic manipulation and selection/expansion of colonies with a hESC-like phenotype (Takahashi et al., 2007). Thus, the sources of genetic changes in hiPSCs include the cell of origin, the reprogramming process and culture adaptation (Ronen and Benvenisty, 2012).

The first observation of recurrent karyotypic changes in hESCs, involving gains of chromosomes 17q and 12 (Draper et al., 2004), which are also commonly observed in embryonal carcinoma cells (the stem cells of malignant germ cell tumours termed teratocarcinomas), prompted the notion that culture adaptation may resemble transformation to malignancy in cancer cells (Baker et al., 2007; Harrison et al., 2007). Studies that followed highlighted additional frequent aberrations, including gains of (parts of) chromosomes 1, 20 and X (Buzzard et al., 2004; Inzunza et al., 2004; Maitra et al., 2005; Mitalipova et al., 2005; Spits et al., 2008). Several groups also raised a possibility that the culture conditions and passaging methods may influence the stability of hPSC karyotypes (Brimble et al., 2004; Buzzard et al., 2004; Caisander et al., 2006; Mitalipova et al., 2005). These observations have prompted large-scale international efforts to generate a more comprehensive catalogue of the mutational spectrum present in the hPSCs and interrogate their possible relation to culture techniques. The International Stem Cell Initiative (ISCI) study screened 120 hESC lines and 11 hiPSC lines from 38 different laboratories at an early and late passage in culture (Amps et al., 2011), whereas Taapken et al. (Taapken et al., 2011) analysed 40 hESC and 219 hiPSC lines from 29 laboratories. One of the first lessons gleaned from these large-scale studies is that hESCs are commonly diploid soon after derivation and can retain a normal karyotype after many passages (for example, in the ISCI study 66% of lines remained normal). On the other hand, a sizable proportion of cell lines examined did show an abnormal karyotype (34% or 42/125 cell lines tested in the ISCI study and 12.9% or 150/1,163 cultures karyotyped in the study by (Taapken et al., 2011)), demonstrating that the occurrence of chromosome variations (numerical and structural) is a common feature of hPSC culture. This is particularly true for later passage cultures, which were approximately twice as likely as early passage cultures to contain cells with abnormal karyotypes (Amps et al., 2011). Cells passaged by enzymatic methods were more likely to acquire genetic abnormalities than the cells passaged manually (Amps et al., 2011), but variants did occur in manually passaged cultures, and the apparent greater stability following manual passaging may be a function of population size, rather than an intrinsic effect on mutation rate or selective advantage (Olariu et al., 2010). There was no difference in the number of abnormalities detected after culturing cells on mouse embryonic fibroblasts versus culturing the cells on Matrigel (Taapken et al., 2011).

With respect to the genomic regions commonly affected in hPSCs, the large-scale studies echoed the earlier individual reports showing non-random changes of chromosomes. For example, of the abnormal hESC karyotypes in the ISCI study, approximately 60% had involvement of at least one of chromosomes 1, 12, 17 and 20 (Amps et al., 2011). Overall, gain of chromosome material is markedly more common than loss in culture adaptation of hPSCs. Recurrent deletions (10p, 18q and 22q) do occur, but their significance remains to be elucidated (Amps et al., 2011; Laurent et al., 2011). Comparison of genetic changes found in hiPSCs versus the ones in hESCs highlighted some similarities but also profound differences between two pluripotent cell types. Similar to hESCs, the most common genetic alteration in hiPSCs is trisomy 12 (Amps et al., 2011; Mayshar et al., 2010; Taapken et al., 2011). However, trisomy 17 which is frequently observed in hESC cultures is rarely seen in hiPSCs (Amps et al., 2011; Ben-David et al., 2011; Mayshar et al., 2010; Taapken et al., 2011). On the other hand, gains of chromosome 8 are more frequent in hiPSC than hESCs (Taapken et al., 2011). Despite these differences, the overall frequency of chromosomal anomalies was similar between hESC and hiPSC cultures (around 13% for both cell types) (Taapken et al., 2011).

## DETERMINING THE CAUSATIVE MUTATIONS IN CULTURE ADAPTATION OF hPSCs

A useful method for pinpointing a minimal region of interest and potentially gene(s) is the occurrence of recurrent chromosome changes, and particularly recurrent chromosome breakpoints. For example, the translocation between chromosomes 9 and 22 (Philadelphia chromosome) (Nowell and Hungerford, 1960; Rowley, 1973) led to identification of the BCR/ABL1 gene fusion (de Klein et al., 1982), which is created at the join between the two chromosomes and is now used for highly successfully targeted therapy in chronic myeloid leukemia. In hPSCs, a recurrent amplification of the 20q11.21 band has been reported in a number of studies, both kaytoypically and sub-karyotypically (Amps et al., 2011; Elliott et al., 2010; Laurent et al., 2011; Lefort et al., 2008; Narva et al., 2010; Spits et al., 2008; Werbowetski-Ogilvie et al., 2009). This has now been narrowed down to a minimal region of about 500 kb that contains thirteen annotated genes, only three of which are expressed in hPSCs: *HM13*, *ID1* and *BCL2L1* (Amps et al., 2011). BCL2L1 has two isoforms, BCL-XL and BCL-XS, but the former is predominant in hPSCs. The known anti-apoptotic role of BCL-XL isoform (Boise et al., 1993) made this gene the prime candidate as a driver mutation in the 20q11.21 region. Indeed, in mixing experiments of normal cells with cells overexpressing any of the three candidate genes from the region (*HM13*, *ID1* or *BCL-XL)*, only *BCL-XL* provided cells with a selective advantage, and this effect was diminished upon knocking down the BCL-XL in cells with the amplified 20q11.21 region (Avery et al., 2013).

The functional proof of *BCL2L1*’s involvement in culture adaptation was greatly facilitated by a relatively small size of the minimal amplicon on chromosome 20, narrowing down the potential pool of candidate genes. Intriguingly, equivalent minimal amplicons have not been identified for other commonly gained chromosomes. Although there are recurrent chromosome regions gained (the long arm of chromosome 1, the short arm of chromosome 12, and the long arm of chromosome 17 with a potential minimal amplicon at the terminal 17q25 band) identified in the hPSCs, these are all substantially sized regions (approximately 118, 34, and 11 Mb respectively; Ensembl) and the breakpoints are not specific to individual chromosome bands (the breakpoint for the chromosome 12 amplicon is centromeric). The use of SNP analysis for detection of sub-karyotypic changes less than 5 Mb in size did not provide any further narrowing down of possible candidate genes/loci. This appears in some respects discouraging, though this scenario is not an uncommon feature of chromosome changes in cancers. For instance, chromosome 8 is frequently gained, as a whole chromosome in leukemias (e.g. found in 15%–20% of myelodysplastic syndrome) (Mitelman, 2014) and as yet no gene of interest has been identified. The absence of clear minimal amplicons on chromosomes other than 20 may reflect the need for several genes/pathways to be altered at once in order to bring about the selective advantage. Although we are yet to demonstrate this possibility in the case of hPSCs, examples from less complex model organisms support this hypothesis. For instance, in *Candida albicans* resistance to antifungal compound fluconazole is acquired through gaining additional copies of the left arm of chromosome V, which harbours two target genes acting independently but in an additive manner to provide cells with the resistance phenotype (Selmecki et al., 2006; Selmecki et al., 2008).

Notwithstanding the need for further refinement of candidate loci by genetic mapping, it is tempting to speculate on candidate genes in addition to *BCL2L1* that may be involved in culture adaptation. A region of chromosome 12 that is frequently amplified in hPSCs harbors several genes related to pluripotency, including *NANOG*, *DPPA3* and *GDF3*, as well as cell cycle regulators such as *CCND2*. It is of note that chromosome 12p also contained *KRAS* which is an oncogene abundantly expressed in testicular germ cell tumors as well as a number of other cancer types (Alagaratnam et al., 2011). Its homolog *ERAS* is highly expressed in mouse ESCs and had been shown to promote tumor like property during teratoma formation (Takahashi et al., 2003). A likely candidate on chromosome 17q is an anti-apoptotic gene *BIRC5* (SURVIVIN). Genetic and pharmacological inhibition of BIRC5 expression caused increased apoptosis of hPSCs *in vitro* and in the teratomas (Blum et al., 2009).

## THE ROLE OF ANEUPLOIDY IN CULTURE ADAPTATION OF hPSCs: EVADING THE SELECTIVE PRESSURE(S)

**Figure 1:**
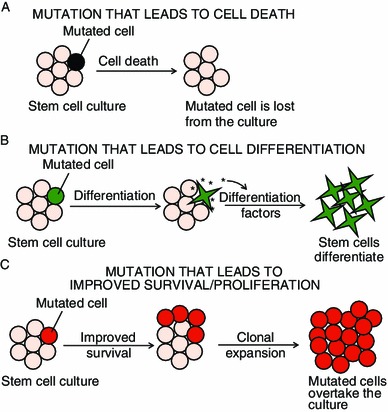
**Possible scenarios of hPSC fates following an acquisition of random mutations**. (A) If a detrimental mutation arises, the mutated cell will die and the mutation will not be propagated in culture. (B) If a mutation causes cell differentiation, the mutant cell will not only be lost from the stem cell pool, but it may also start producing factors that induce differentiation of other hPSCs. Due to the loss of stem cells through differentiation, the mutated cell will not be selected for and its progeny will be eventually lost from the culture. (C) If a mutation causes improved survival and/or increased proliferation, the mutated cell is likely to outcompete its neighboring cells and populate the culture with its own progeny

For a randomly mutated hPSC to persist and eventually populate the entire culture with its own progeny, the acquired mutation must allow the cell to break away from the normal regulation of stem cell fates, i.e. self-renewal, differentiation and death. For example, a mutant cell and its progeny have to either proliferate more rapidly than the neighboring cells or they have to be less prone to cell death and differentiation, as either death or differentiation would lead to an irreversible loss of stem cells (Fig. 1). In a set of experiments mimicking the occurrence of sporadic mutants within a sea of normal cells, Olariu et al. found that aneuploid hPSCs with representative commonly observed karyotypic changes quickly outcompeted their normal counterparts (Olariu et al., 2010). One of the major hallmarks of adapted hPSCs is an improved ability to create stem cell colonies following replating as single cells in cloning efficiency assays (Enver et al., 2005; Harrison et al., 2007). Time-lapse tracking of two different euploid lines and their aneuploid counterparts has revealed at least three specific bottlenecks restricting the colony expansion of normal cells which were alleviated in the adapted cells to a large extent: (i) survival post-plating, (ii) the ability of cells to re-enter the cell cycle, and (iii) survival of daughter cells following mitosis (Barbaric et al., 2014). Anti-apoptotic mechanisms are obvious candidates for allowing mutant cells to progress though normally restrictive bottlenecks. Indeed, characterization of *BCL2L1* as a driver mutation on chromosome 20q is consistent with this notion (Avery et al., 2013), and a number of studies showed a reduced apoptotic response of culture adapted cells (Avery et al., 2013; Herszfeld et al., 2006; Yang et al., 2008). Nonetheless, the links between culture adaptation and apoptosis appear to be multifaceted. When Harrison et al. stimulated extrinsic and intrinsic apoptotic pathways in the normal and culture adapted cells, it was the adapted cells that showed higher sensitivity to apoptosis (Harrison et al., 2009). These observations support the notion that culture adaptation may arise through various routes, as a result of different selection pressures operating under specific culture conditions (Harrison et al., 2009).

In addition to apoptosis, the loss of stem cells in culture can arise through differentiation. Thus, culture adaptation could be selecting against the ability of stem cells to differentiate. In an extreme scenario, this may result in a “nullipotent” state in which cells may self-renew but no longer differentiate, as is the case with many human embryonal carcinoma cell lines (Andrews et al., 1980; Matthaei et al., 1983). The ability of hPSCs to differentiate is a crux of many potential applications of these cells, and changes in differentiation ability would not only affect efficient production of differentiated cell types for cell therapy, but could also hamper their application in disease modeling and drug discovery (Goldring et al., 2011). Culture-adapted cells appear capable of extensive differentiation, but their differentiation deviates from their normal counterparts (Fazeli et al., 2011; Werbowetski-Ogilvie et al., 2009). For example, cells with a gain of chromosome 20q11.1-11.2 had a significantly reduced propensity to differentiate down the hematopoietic lineage and in the neural differentiation experiments the variant cells displayed more immature phenotypes along the differentiation trajectory than their wild-type controls (Werbowetski-Ogilvie et al., 2009). For some adapted cell lines genetic changes resulted in the repression of extraembryonic endoderm differentiation (Fazeli et al., 2011). Given that endoderm cells produce factors such as Bone Morphogenetic Proteins (BMPs), which in turn induce differentiation of hPSCs (Pera et al., 2004; Xu et al., 2002), selection against spontaneous endoderm differentiation might provide a mechanism for a selective advantage (Fig. 1).

## PARALLELS OF GENETIC CHANGES IN hPSCs WITH HUMAN GERM CELL TUMOURS AND OTHER CANCERS

A selective growth advantage imparted by an acquired mutation is at the core of cancer development. Thus, the process of culture adaptation of hPSCs is reminiscent of malignant cell transformation. The recurrent mutations in hPSCs are similar to the ones observed in testicular germ cell tumours (TGCT) (Baker et al., 2007; Draper et al., 2004; Harrison et al., 2007). Human TGCT are typically aneuploid with multiple chromosomal rearrangements (Oosterhuis et al., 1990). Seminomas, which constitute nearly half of all TGCT, typically have a near 4N DNA content, whereas nonseminomatous TGCT have nearer a 3N constitution, leading to the suggestion that these tumours originate from a tetraploid germ cell, with subsequent chromosomal rearrangement and loss during tumour progression. We previously suggested that abnormalities in the control of the switch between mitosis and meiosis in primordial germ cells as they populate the male genital ridge during embryonic development might underlie the origins of aneuploidy in TGCT (Adamah et al., 2006). Although hPSCs acquire aneuploidy with respect to a few chromosomes, they rarely reach the gross aneuploid state of typical human embryonal carcinoma cells with a near triploid chromosome number including multiple rearrangements (Wang et al., 1980). An exception is the hESC line, CH-ES1, derived from a blastomere of a cleavage stage embryo and containing multiple chromosomal rearrangements (Hovatta et al., 2010). Among the variant chromosomes of human embryonal carcinoma cells, a gain of the short arm of chromosome 12, typically as an isochromosome of 12p, is almost always present in invasive tumours but less is often found in carcinoma *in situ*, suggesting that this gain contributes to cancer progression but not initiation (Atkin and Baker, 1982; Oosterhuis et al., 1990). Gains of chromosome 17 and of chromosome 20 are also reported (Amps et al., 2011; Avery et al., 2013; Skotheim et al., 2002). Studies of minimal amplicons of chromosome 12p have suggested that a region that includes NANOG, at 12p13.31 may be the critical region (Korkola et al., 2006), though other regions such as 12p11.2-p12.1 have also been identified (Zafarana et al., 2003).

Although karyotypic changes in hPSCs seem to parallel the ones found in TGCTs, it is worth noting that similar mutations appear in other cancers. Chromosome 17 gain is a common change in many cancers, and isochromosome of the long (q) arm is suggestive of an aggressive clinical course (Atlas of Chromos Cancer). Chromosome 17 is also found in numerous recurrent translocations in leukemias and solid tumours, such as the translocations with chromosomes 15 and 22 found in acute promyelocytic leukemia and dermatofibrosarcoma protuberans, respectively. Similarly, trisomy 12 is a common finding (16%) in chronic lymphocytic leukemia (Dohner et al., 2000). Chromosome 1 rearrangements resulting in duplication of the part or all of the long arm as seen in hPSCs are found in up to 26% of abnormal multiple myeloma cancers. Rearrangements of chromosome 20 observed in hPSCs are less frequently seen in cancers. Interestingly, there is one report of a translocation between chromosomes 20 and 6, resulting in a gene fusion between *BACH2* and *BCL2L1* leading to overexpression of *BCL2L1*, the candidate gene in the 20q11.21 amplicon identified in hPSCs (Turkmen et al., 2011). This fusion was found in a cell line from a patient with relapsed high-grade B-cell lymphoma, which showed a particularly aggressive course.

A key question that arises from the observed similarities between genetic changes in hPSCs and cancer cells is whether adapted hPSCs exhibit similar malignant properties to cancer. Hanahan and Weinberg summarized the hallmarks of cancer into the following categories: (i) self-sufficiency in growth signals, (ii) insensitivity to anti-growth signals, (iii) evasion of apoptosis, (iv) sustained angiogenesis, (v) tissue invasion and metastasis, and (vi) unlimited replicative potential (Hanahan and Weinberg, 2011). Unlimited replicative potential *in vitro* is a defining property of karyotypically normal hPSCs, but additional hallmarks of cancer cells have been noted in hPSCs with aneuploidy. For example, a hPSC line with a gain of 20q11.1-11.2 showed signs of self-sufficiency in growth signals, as it retained undifferentiated phenotype even in the absence of basic fibroblast growth factor which is normally necessary for self-renewal of hPSCs (Werbowetski-Ogilvie et al., 2009). Evasion of apoptosis as noted in some culture adapted hPSCs is also reminiscent of the key hallmarks of cancer (Avery et al., 2013; Herszfeld et al., 2006; Yang et al., 2008). For purposes of cell replacement therapies, a particularly concerning feature of culture adapted cells is the ability to form teratocarcinomas—tumors that in addition to differentiated derivatives contain a remnant pool of undifferentiated stem cells, which can be placed back into culture and grown as hPSCs (Andrews et al., 2005; Blum and Benvenisty, 2009). As it is unlikely that undifferentiated hPSC will be used to transplant into patients, further rigorous testing is warranted to establish whether any of the mutations in culture adapted hPSCs also confer a growth advantage on their differentiated progeny.

## MECHANISMS OF ANEUPLOIDY IN hPSCs, CANCER AND PREIMPLANTATION EMBRYOS: THE ROLE OF SPINDLE ASSEMBLY CHECKPOINT

Generation of mutations is an essential prerequisite for culture adaptation. When considering mechanisms of mutations in hPSCs, it is worth noting the differences in how the recurrent chromosome changes occur. For example, chromosome 12 is mostly gained as a whole chromosome or as an isochromosome, whilst chromosome 1 and 17 are gained via structural rearrangements such as intra-chromosomal duplication and unbalanced translocations involving other chromosomes in addition to trisomy and isochromosome formation (Amps et al., 2011; Baker et al., 2007). This suggests that chromosome 12 anomalies are more commonly mitotic nondisjunction errors whilst the abnormalities of chromosomes 1 and 17 may reflect their comparative richness in repetitive DNA sequences (e.g. segmental duplications) and occur via non-homologous recombination events using these repetitive sequences and mis-repair of DNA breaks.

Changes in chromosome numbers are often caused by errors in separation of sister chromatids during mitosis. A key regulatory mechanisms controlling accurate chromosome segregation to daughter cells is the spindle assembly checkpoint (SAC) (also known as the mitotic checkpoint), with conserved components from yeast to man (Musacchio and Salmon, 2007). The major SAC components are encoded by *MAD* (mitotic-arrest deficient) genes *MAD1*, *MAD2* and *MAD3* (BUBR1 in humans), the *BUB* (budding uninhibited by benzimidazole) gene *BUB1*, and *AURORA KINASE B (Ipl1 in S. cerevisiae)* (Musacchio and Salmon, 2007). SAC becomes active as cells enter prometaphase, when it monitors the interaction between kinetochores and microtubules. Once all kinetochores have attached to the bipolar spindle with equal tension, the SAC is inactivated and cells are allowed to proceed through mitosis (Musacchio and Salmon, 2007).

A number of studies have demonstrated that dysregulation of SAC function perturbs various aspects of mitosis and leads to the formation of aneuploid cells (Holland and Cleveland, 2009). SAC components are commonly enriched in cancer cells and have been linked to their active cell cycle and unstable karyotypes. Overexpression or knock-down of SAC proteins, such as MAD2, BUB1 and AURORA KINASE B causes premature separation of sister chromatids, chromosome bridging, and in some cases, cytokinesis failure (Duijf and Benezra, 2013). If cells that have gone through abnormal mitosis enter the next cell cycle, they often activate the p53 dependent apoptosis pathway (Thompson and Compton, 2010). A recent study by Crasta et al. (2012) showed that in HeLa cells, micronuclei generated due to mitotic chromosome segregation errors, contained whole chromosomes that had persistent albeit aberrant DNA replication (Crasta et al., 2012). Defective DNA replication and damage can lead to mutagenesis or chromosome pulverization within the micronuclei. As cells continue to divide, when the nuclear envelop breaks down, micronuclei in a subset of cells may join the mitotic chromosomes and reintegrate into the nucleus of daughter cells (Crasta et al., 2012). The reincorporation of fragmented micronuclei from missegregated chromosomes may give rise to chromothripsis, the phenomenon that involves complex genome rearrangements in a limited genomic region after one single catastrophic event during cell cycle (Jones and Jallepalli, 2012).

In addition to cancer cells, human oocytes are particularly prone to chromosome segregation errors during meiosis (Hassold and Hunt, 2001). Although SAC functions to some extent in mammalian oocytes, it is insufficient to detect or correct unaligned chromosomes. This in turn renders oocytes innately susceptible to aneuploidy, which is made worse by an age-related reduction in key SAC regulators and factors that maintain chromosome and spindle structure (Jones and Lane, 2013; Sebestova et al., 2012). Chromosome segregation errors also happen frequently post fertilization in mammalian preimplantation embryos. Immunostaining studies revealed multipolar division marked by multiple spindle poles (Chatzimeletiou et al., 2005). Genome-wide assessment of copy number variations (CNV) and single nucleotide polymorphisms (SNPs) enabled detailed identification of chromosome region gain, deletion and aneuploidy. BAC array based cytogenetic study of 3–4 day human IVF embryos (mostly at 8-cell stage) found higher incidence of aneuploidy than those found in early pregnancy or at birth (Vanneste et al., 2009). This discrepancy may account for pregnancies lost before their detection. Both cleavage and blastocyst stage IVF embryos also showed frequent incidence of mosaic aneuploidy, with a subset of blastomeres containing abnormal number of chromosomes. Overall, these observations suggested that mammalian oocytes and preimplantation embryos may have a somewhat “leaky” SAC machinery due to their unique cell cycle profiles and genes specifically expressed in the gametes. The critical selection against chromosome abnormalities does not appear to occur until the time of implantation or shortly after, with mitotic aneuploidy typically affecting significant proportion of human IVF embryos (Fragouli et al., 2013; Fragouli et al., 2008; Fragouli and Wells, 2011; Vanneste et al., 2009). Despite the prevalence of mosaic aneuploidy, hESCs derived from IVF blastocysts are mostly karyotypically normal, suggesting that aneuploidy may act as a negative selection barrier for hESC derivation.

The molecular mechanisms of chromosome segregation that leads to aneuploidy in hPSCs are poorly characterised. The cell cycle of hPSC is relatively fast compared to many somatic cells (Becker et al., 2006), and high expression of SAC components may play a role in aberrant divisions as was observed in cancer cells. Indeed, microarray studies revealed that similar to cancer cells and preimplantation embryonic cells, several SAC proteins such as MAD2 and BUB1 are highly expressed in undifferentiated hESC and are down-regulated upon differentiation (data obtained from www.amazonia.transcriptome.eu). It also appears that in contrast to differentiated cells, in which SAC triggers apoptosis as a response to microtubule poison-generated polypoidy, SAC in mouse and hPSCs is uncoupled from apoptosis (Mantel et al., 2007).

**Figure 2:**
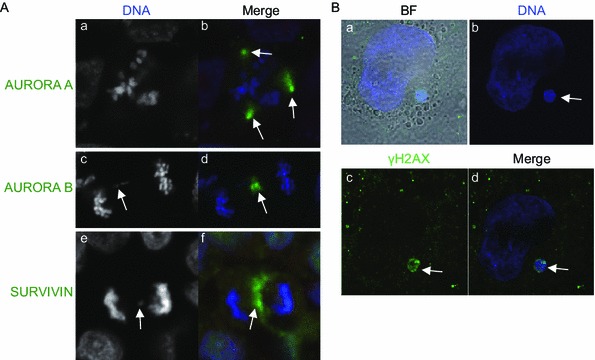
**Aberrant mitosis and micronucleus formation in hPSCs**. (A) Immunostaining of euploid HUES1 hESCs for AURORA A (a and b), AURORA B (c and d) and SURVIVIN (e, f). Nuclei are counterstained with DAPI. Panels (a and b) show an example of a multipolar division with multipolar spindles. Note that AURORA A (green staining) is localized to all three spindle poles (white arrows in b). Panels c–f show an example of a chromosomal bridge (white arrows in c and e). Note that AURORA B (d, green staining) and SURVIVIN (f, green staining) are concentrated at the cleavage furrow and the lagging kinetochore (white arrows in d and e). (B) Micronucleus formation (white arrow) in H9 hESCs after inhibition of AURORA B with 50 nm of AZD1152 for 24 h and release from the inhibitor for 24 h. Micronucleus stained positive for γH2AX, a marker of DNA damage (green)

**Figure 3:**
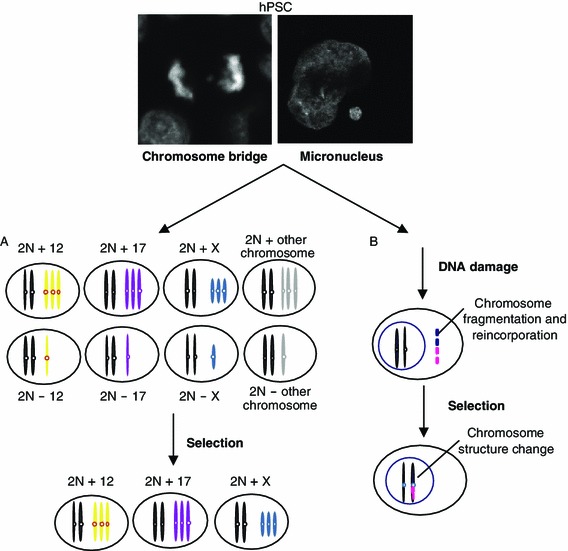
**Schematic view depicting putative mechanisms of chromosome number and structure changes due to aberrant mitosis**. (A) A chromosome bridge can lead to unequal segregation of sister chromatids into two daughter cells. Gains of certain chromosomes, such as 12, 17 and X may give cells survival advantage under stressful conditions. On the other hand, loss of those chromosomes or gains of other chromosomes, that are either neutral or not compatible with cell survival, may lead to cell death/differentiation. During subsequent passaging *in vitro*, progeny of cells with gained chromosome 12, 17 and X are likely to persist and overtake the culture. (B) Alternatively, lagging chromosomes formed during aberrant mitosis may form micronuclei. In the process termed chromothripsis, the genetic material within the micronucleus may become fragmented and then reintegrated into the nucleus, resulting in chromosome structure change and gene copy number variation

We found that hPSCs highly express many SAC proteins that are also enriched in oocytes and preimplantation embryos, and can undergo abnormal mitosis during routine culture (Fig. 2A). Moreover, inhibition of SAC function led to the formation of micronuclei, which expressed DNA damage marker γH2AX (Fig. 2B). These observations demonstrated that hPSCs are indeed susceptible to erroneous mitotic division, and warrant in-depth analysis of frequency of their occurrence and underlying molecular mechanisms. Based on well documented large-scale cytogenetic characterizations of hPSCs and parallel findings from cancer studies, we propose a model to explain recurrent karyotype changes found in hPSC (Fig. 3): disturbing the function of SAC components or mitosis machinery leads to defective cell division with missegregated whole or partially broken chromosomes and/or the formation of micronucleus with extensive DNA damage and fragmentation. For many of the cells, such changes will prove detrimental and result in cell death. By chance, in a very small proportion of mutated cells the combination of genes affected will be such that it endows them with survival advantage and preferential retention during prolonged passaging. However, such adaptation may hamper differentiation of hPSCs and potentiate them to gain additional oncogenic genetic changes.

## FUTURE PERSPECTIVES

Although studies to date have revealed a plethora of genetic changes in hPSCs, the true extent of genetic variation in hPSCs is only likely to become apparent when we embark on whole genome sequence analysis. The notion of acquiring whole genome sequence of a significant number of cells from each culture condition may seem like a formidable task at the moment, but given the rapid progress of the sequencing technology and the concomitant drop in price, it is conceivable that this type of analyses will become routine in the near future. Nonetheless, the greater challenge will be decoding the extraordinary breadth of the sequence data in the quest to determine which of the numerous mutations in the genome are causative and which are simply passenger events (Stratton, 2011). Indeed, a decade since the first report of the recurrent chromosomal aberrations in hPSCs (Draper et al., 2004) and a number of suggested candidate genes (Amps et al., 2011; Laurent et al., 2011) only one gene, *BCL2L1*, has been definitively shown to confer growth advantage to the variant cells (Avery et al., 2013).

The advent of highly sensitive methods for mutation detection raises a provocative question whether *any* mutation detected should preclude the use of cells in various *in vitro* applications and particularly in potential therapies. It is also important to consider whether a mutation that does not affect the phenotype and behavior of hPSCs may have harmful effects once the cells are differentiated to a particular lineage of interest and/or transplanted into a different niche *in vivo*. Clearly, future efforts should be directed to develop predictive tests of functionally important mutations. As daunting as this task may seem, it will be of fundamental importance for ensuring therapeutic safety of hPSCs. Particularly informative should be investigations of mechanisms that lead to genetic changes in hPSCs as they may reveal inherent weaknesses of mutant cells that could make them amenable to therapeutic targeting. For example, an intriguing possibility is that aneuploidy triggers common transcriptome changes (Torres et al., 2007), which would allow targeted ablation of mutant cells. Such approach was recently demonstrated in the case of trisomic mouse embryonic fibroblasts and aneuploid cancer cells, which were successfully eliminated from cultures by small compounds that exerted antiproliferative effects only on aneuploid cells but not their karyotypically normal counterparts (Tang et al., 2011). In summary, improved ability to detect mutations and assess their functional significance, coupled with targeted approaches to eliminate abnormal cells from cultures will ensure positive prospects for translation of hPSCs research into the clinic.

## ABBREVIATIONS

CNV: copy number variation
hESCs: human embryonic stem cells
hiPSCs: induced pluripotent stem cells
hPSCs: human pluripotent stem cells
IVF: in vitro fertilisation
SAC: spindle assembly checkpoint
SNP: single nucleotide polymorphism
TGCT: testicular germ cell tumour
